# Perinatal Deaths: Autopsy Findings and Medico-Legal Evaluation from a Forensic Medicine Perspective

**DOI:** 10.3390/diagnostics16152341

**Published:** 2026-07-27

**Authors:** Sertac Dalgic, Abuzer Gulderen, Murat Kamalak, Ibrahim Uzun

**Affiliations:** 1 Akhisar Forensic Medicine Branch Office, The Council of Forensic Medicine, 45000 Manisa, Türkiye; 2 Gaziantep Forensic Medicine Group Presidency, The Council of Forensic Medicine, 27000 Gaziantep, Türkiye; md.abuzergulderen@gmail.com (A.G.); mkamalak@outlook.com (M.K.); 3 1st Forensic Medicine Specialization Department, The Council of Forensic Medicine, 34000 Istanbul, Türkiye; ibrahim.uzun@iuc.edu.tr

**Keywords:** perinatal deaths, forensic medicine, autopsy, stillbirth

## Abstract

**Background**: Perinatal deaths remain a major public health concern and present unique medico-legal challenges. Forensic autopsy plays a crucial role in determining the cause of death, distinguishing live birth from stillbirth, assessing fetal viability, and identifying traumatic or non-natural causes of death. **Methods**: This retrospective study included all perinatal cases referred to the Gaziantep Forensic Medicine Group Presidency between 2021 and 2024. Demographic characteristics, gestational age, autopsy findings, histopathological and toxicological examinations, hydrostatic lung test results, and final forensic diagnoses were evaluated. **Results**: Seventeen of 339 infant forensic cases met the inclusion criteria. Fourteen underwent complete forensic autopsy, whereas three were evaluated by external postmortem examination and medical records. Despite a multidisciplinary forensic investigation that included external examination, complete forensic autopsy, systematic macroscopic organ evaluation, routine histopathological examination, hydrostatic lung flotation testing, toxicological analysis when indicated, review of maternal medical and obstetric records, judicial investigation records, and placental and umbilical cord histopathology whenever available, no definitive cause of death could be established in six cases. Advanced decomposition prevented determination in two cases. One case was classified as an undiagnosed natural death, whereas no specific cause of death could be assigned in the remaining three cases. Placental examination was available in only a minority of cases, whereas umbilical cord histopathology was performed in nearly all autopsied cases. Causes of death included traumatic injury, pulmonary immaturity, intrauterine infection, congenital anomalies, maternal drug exposure, placental abruption, maternal disease, and umbilical cord pathology. **Conclusions**: Perinatal death investigations require a multidisciplinary forensic approach integrating autopsy, histopathology, toxicology, placental examination, and clinical information. Beyond determining the cause of death, forensic evaluation is essential for assessing live birth, fetal viability, suspected abuse, maternal violence, medical malpractice, and criminal investigations.

## 1. Introduction

Perinatal mortality remains a major global public health concern despite substantial advances in prenatal care, obstetric management, and neonatal intensive care. According to the World Health Organization (WHO), approximately 2 million stillbirths occur worldwide each year, and the majority are reported in low- and middle-income countries [1]. In addition to stillbirths, early neonatal deaths continue to contribute significantly to perinatal mortality, reflecting disparities in healthcare access, maternal health, and socioeconomic conditions [2]. Consequently, reducing perinatal mortality has become a global priority for improving maternal and child health outcomes [3].

Beyond its public health significance, perinatal death represents a unique challenge in forensic medicine. Establishing whether a fetus or newborn was born alive, determining the timing and mechanism of death, identifying traumatic injuries, and differentiating natural from non-natural deaths are fundamental objectives of the forensic investigation [4]. These evaluations become particularly important in cases involving suspected infanticide, concealed pregnancy, abandonment, maternal trauma, substance abuse during pregnancy, or allegations of medical malpractice [5]. In such situations, forensic examination provides essential scientific evidence for judicial authorities while also contributing to public health surveillance and preventive strategies [6].

A comprehensive forensic evaluation of perinatal death requires integration of scene investigation, maternal medical history, obstetric records, external examination, complete autopsy, histopathological analysis, toxicological investigations, microbiological testing when indicated, and careful examination of the placenta and umbilical cord [7]. Among these, the hydrostatic (lung flotation) test remains a valuable adjunctive tool for assessing pulmonary aeration, although its findings should always be interpreted together with autopsy, histopathological, and circumstantial evidence [8]. Similarly, placental and umbilical cord examinations frequently provide critical information regarding fetal hypoxia, intrauterine infection, vascular compromise, and other pathological processes contributing to fetal or neonatal death [9].

Although numerous studies have investigated the epidemiology and pathological causes of perinatal mortality, relatively few have focused specifically on its medico-legal aspects [10]. Publications integrating autopsy findings with forensic interpretation, judicial investigation, and legal implications remain limited, particularly in developing countries where medico-legal practices may differ substantially. Therefore, retrospective analyses of forensic perinatal cases provide valuable information for both forensic pathologists and clinicians involved in perinatal death investigations [11].

The present study aimed to retrospectively evaluate perinatal deaths referred for forensic examination by analysing demographic characteristics, medico-legal circumstances, autopsy findings, histopathological and toxicological results, and final forensic diagnoses. Few published studies have comprehensively evaluated perinatal deaths from a forensic medicine perspective by integrating autopsy findings with judicial investigations, forensic decision-making, toxicological analyses, placental pathology, and medico-legal interpretation. Therefore, the present study aims to address this gap by highlighting the practical role of forensic autopsy in judicial investigations of perinatal deaths rather than merely describing pathological findings. In addition, this study discusses the role of forensic investigation in distinguishing live birth from stillbirth, determining the cause and manner of death, and contributing to judicial decision-making in perinatal deaths.

## 2. Materials and Methods

### 2.1. Study Design and Case Selection

This retrospective descriptive case series was conducted at the Gaziantep Forensic Medicine Group Presidency, Türkiye. The study included all perinatal death cases referred for forensic postmortem examination between January 2021 and December 2024.

During the study period, a total of 339 forensic infant death cases were reviewed. Cases were eligible if they met the World Health Organization definition of the perinatal period (stillbirths and early neonatal deaths) and had sufficient medico-legal documentation. Seventeen cases fulfilled the inclusion criteria and were included in the final analysis. Cases with incomplete records or those falling outside the perinatal period were excluded.

### 2.2. Data Collection

Demographic and medico-legal data were retrieved retrospectively from forensic case files, autopsy reports, judicial investigation records, hospital medical records, and laboratory reports.

The following variables were evaluated: sex, nationality, gestational age, birth weight, body length, place and circumstances of delivery, circumstances surrounding death, external examination findings, complete autopsy findings, hydrostatic (lung flotation) test results, histopathological findings, toxicological examination results, placental and umbilical cord examination findings (when available), final forensic diagnosis, and final medico-legal opinion.

### 2.3. Forensic Examination

Fourteen cases underwent complete forensic autopsy according to the national forensic autopsy protocol of the Turkish Council of Forensic Medicine. Three cases underwent external postmortem examination because a complete autopsy was not requested by the judicial authorities.

All autopsies included a detailed external examination followed by systematic internal examination of the cranial, thoracic, abdominal, and cervical cavities. Organ findings were documented according to standard forensic pathology practice.

The hydrostatic (lung flotation) test was performed whenever appropriate to assist in distinguishing live birth from stillbirth. Test results were interpreted together with macroscopic findings, histopathological examination, and case circumstances, rather than being considered in isolation.

### 2.4. Histopathological and Toxicological Examination

Representative tissue samples were collected from the central nervous system (including the cerebral hemispheres, cerebellum, brainstem, and dura mater), heart, lungs, liver, kidneys, adrenal glands, thymus, spleen, placenta, and umbilical cord whenever available.

Routine histopathological examination was performed using hematoxylin and eosin staining. Additional histochemical or immunohistochemical analyses were performed when clinically indicated.

Toxicological analyses were requested whenever maternal drug exposure, poisoning, or suspicious death was suspected. Biological specimens included blood, urine, vitreous humor, gastric contents, and internal organs according to standard forensic toxicology protocols.

### 2.5. Definitions

For the purposes of this study, standardized definitions based on the World Health Organization (WHO) recommendations were used. Perinatal death was defined as fetal deaths occurring at or after 22 completed weeks of gestation (or birth weight ≥500 g when gestational age was unavailable) together with deaths of live-born infants occurring within the first seven completed days of life.

A live birth was defined as the complete expulsion or extraction of a fetus from its mother, irrespective of gestational age, which, after separation, showed any evidence of life such as breathing, heartbeat, pulsation of the umbilical cord, or definite voluntary muscle movement.

A stillbirth was defined as the birth of a fetus showing no evidence of life after complete expulsion or extraction from the mother and meeting the WHO criteria for the perinatal period.

Because the concept of viability varies among legal systems, viability in the present forensic study was assessed individually based on gestational age, fetal growth, developmental maturity, autopsy findings, and all available clinical and investigative information, rather than by gestational age alone. In the medico-legal evaluation, fetal viability was interpreted according to Turkish forensic practice while maintaining the WHO definitions for inclusion criteria.

### 2.6. Statistical Analysis

Data were analyzed descriptively using IBM SPSS Statistics (Version IBM SPSS Statistics Version 29.0; IBM Corp., Armonk, NY, USA). Continuous variables are presented as mean ± standard deviation (SD) or median (interquartile range [IQR]), depending on data distribution. Categorical variables are expressed as frequencies and percentages.

Because of the limited sample size, no inferential statistical analyses were performed.

### 2.7. Ethical Approval

This study was approved and permission to access forensic case records was obtained from the Turkish Council of Forensic Medicine, with the approval 10 February 2026 date and 21589509/2026/26 reference number. The study was conducted in accordance with the ethical principles of the Declaration of Helsinki. All data were anonymized before analysis to ensure patient confidentiality.

## 3. Results

During the study period, 339 forensic infant death cases were reviewed. Of these, 17 perinatal deaths fulfilled the inclusion criteria and were included in the study. Among the 17 cases, 11 (64.7%) were male, 5 (29.4%) were female, and 1 (5.9%) was of undetermined sex because of advanced decomposition. Deaths occurred most frequently during summer and autumn (five cases each), followed by winter (four cases) and spring (three cases). Maternal nationality was Turkish in six cases, Syrian in seven, another foreign nationality in one, and could not be established in three cases. The median gestational age was approximately 34 weeks (range: 21–42 weeks). Birth weight ranged from 350 g to 3275 g, whereas body length ranged from 26.5 cm to 53 cm (Table 1). The median gestational age was 32.5 weeks (IQR: 28.0–36.5), the median birth weight was 1966 g (IQR: 1343–2360), and the median body length was 42.0 cm (IQR: 35.0–46.0) (Table 2).

Thirteen infants were delivered preterm, three at term (≥38 weeks), and one post-term (>42 weeks). Among the preterm deliveries, eight were stillbirths, three were live births, and one could not be classified because of advanced decomposition. Of the three term deliveries, one infant was stillborn, one survived for three days after birth, and one could not be evaluated owing to advanced decomposition. The only post-term infant was born alive and died on the first postnatal day. Gestational age ranged from 22 weeks to post-term, and the longest postnatal survival was three days.

Fourteen cases (82.4%) underwent complete forensic autopsy, while three cases (17.6%) underwent external postmortem examination only because complete autopsy was not requested by the judicial authorities. Histopathological examination was performed in all autopsied cases. Toxicological analysis was requested whenever clinically or legally indicated.

Hydrostatic (lung flotation) testing demonstrated findings consistent with live birth in four cases (23.5%), whereas eight cases (47%) showed non-aerated lungs compatible with stillbirth or intrauterine fetal death. In two advanced decomposition cases, pulmonary findings could not be reliably interpreted. The remaining three cases underwent external postmortem examination only (Table 3).

Standard anthropometric assessment was completed in all cases and included body weight, crown-heel length, sitting height, head circumference, chest circumference, abdominal circumference, and foot length to evaluate fetal growth and gestational maturity.

Comprehensive toxicological and histopathological analyses were performed in all autopsied cases. Amphetamine and methamphetamine were detected in two infants. In one case, the cause of death was attributed to transplacental intoxication with stimulant drugs. In the second case, amphetamine, methamphetamine, and phenprobamate were identified; however, advanced decomposition precluded determination of both live birth status and the cause and mechanism of death.

Placental tissue accompanied five autopsied cases and was submitted for histopathological examination in all instances. No placental tissue was available in the remaining nine autopsy cases. Histopathological examination of the umbilical cord was performed in 13 cases and was unavailable in one.

The lungs were routinely removed en bloc with the cervicothoracic organs in all autopsied infants, followed by gross examination, hydrostatic lung flotation testing, and histopathological evaluation. Four cases demonstrated pulmonary aeration with positive hydrostatic flotation tests, whereas ten showed negative flotation tests consistent with unventilated lungs. All autopsied live-born infants demonstrated aerated lungs and positive flotation tests (Table 4). Among intrauterine deaths, eight autopsied cases demonstrated unventilated lungs and negative flotation tests. The remaining two intrauterine deaths were evaluated without autopsy. Live birth status could not be determined in two severely decomposed cases.

Regarding the place of delivery or recovery, seven infants were delivered in hospital, three at home, one in a taxi, and one on the street. Two infants were recovered inside bags, one was discovered in the courtyard of a mosque, one was recovered after burial, and one died in utero following maternal death. In two hospital stillbirths delivered by cesarean section, the families had filed formal complaints alleging medical malpractice.

Autopsy was performed in 14 of the 17 perinatal cases. Final forensic opinions for these cases were issued by the First Board of Specialization of the Council of Forensic Medicine. In the remaining three cases, conclusions were reached without a full autopsy based on external postmortem examination, medical records, and investigative documentation.

The final forensic investigation identified a broad spectrum of causes of death (Table 5). These included blunt head trauma, pulmonary immaturity associated with prematurity, intrauterine pulmonary infection, maternal amphetamine/methamphetamine exposure, placental abruption secondary to maternal trauma, umbilical cord entanglement, congenital anomalies, maternal cardiac disease, and intrauterine fetal death. In two decomposed cases, neither live birth status nor the cause and mechanism of death could be established.

Special medico-legal circumstances were identified in several cases. Two investigations were initiated following allegations of medical malpractice after hospital delivery. Two cases involved maternal physical assault during pregnancy; in one case, a causal relationship between maternal trauma and fetal death was established, whereas no causal relationship was identified in the other. One case involved transplacental amphetamine/methamphetamine exposure resulting in fatal intoxication. Two abandoned infants and two unidentified infants were also included in the study, highlighting the diverse medico-legal circumstances encountered during forensic investigations of perinatal deaths.

Among the 14 cases evaluated by the First Board of Specialization, two were reported as “unable to determine whether the infant had been born alive; cause and mechanism of death undetermined,” whereas one was classified as “natural death not diagnosed at autopsy.” In three additional cases, the forensic opinion focused primarily on fetal viability and live birth status rather than on the cause of death, concluding that the infant was “non-viable and stillborn,” “died in utero and was delivered stillborn,” or “viable but died in utero before delivery.”

A definitive cause of death was established in eight cases. Causes included blunt cranio-cerebral trauma with skull fracture and intracranial hemorrhage (*n* = 1), respiratory failure secondary to intrauterine pulmonary infection (*n* = 1), transplacental amphetamine-methamphetamine intoxication (*n* = 1), placental abruption following maternal blunt trauma (*n* = 1), respiratory failure due to pulmonary immaturity associated with prematurity (*n* = 1), polyhydramnios associated with congenital anomalies (*n* = 1), intrauterine asphyxia resulting from umbilical cord entanglement (*n* = 1), and intrauterine fetal death following maternal death caused by pregnancy-related decompensation of pre-existing cardiac disease (*n* = 1).

No definitive cause of death was identified in six cases. Advanced decomposition prevented determination in two cases. One case was classified as an undiagnosed natural death, whereas no specific cause of death was assigned in the remaining three cases. Among the three cases evaluated without autopsy, the reported causes of death were neural tube defect (*n* = 1), natural death related to underlying disease (*n* = 1), and intrauterine developmental anomaly associated with pre-existing disease (*n* = 1).

## 4. Discussion

The forensic investigation of perinatal deaths remains one of the most challenging areas of forensic pathology because it requires the integration of autopsy findings, obstetric history, maternal medical conditions, placental pathology, histopathological examination, and judicial investigation [12]. Unlike adult forensic autopsies, the objective extends beyond determining the immediate cause of death and includes establishing fetal viability, differentiating live birth from stillbirth, identifying potentially preventable causes, and evaluating possible criminal or medical liability [13].

In the present study, only 17 of 339 forensic infant deaths met the inclusion criteria for perinatal death. Although the number of cases was relatively small, the series demonstrated considerable heterogeneity regarding gestational age, medico-legal circumstances, pathological findings, and causes of death. This diversity reflects the broad spectrum of conditions encountered during forensic investigation of perinatal deaths and emphasizes the necessity of an individualized multidisciplinary evaluation [14,15].

One of the principal objectives of forensic examination is determining whether a newborn was born alive. In our series, hydrostatic lung testing contributed to the evaluation of pulmonary aeration in most autopsied cases. However, as emphasized in previous studies, the hydrostatic test should never be interpreted in isolation because false-positive and false-negative results may occur in cases of decomposition, artificial ventilation, severe prematurity, or pulmonary disease [16,17]. Therefore, the interpretation of lung flotation findings together with macroscopic examination, histopathology, clinical history, and scene investigation provides a substantially more reliable assessment of live birth than any single diagnostic method [18].

Prematurity and congenital anomalies constituted an important proportion of the identified causes of death in our study [19]. Similar observations have been reported in previous autopsy series, in which pulmonary immaturity, congenital malformations, and intrauterine developmental abnormalities represent major contributors to perinatal mortality [20,21]. These findings emphasize the continuing importance of comprehensive fetal autopsy for confirming prenatal diagnoses, identifying previously unrecognized anomalies, and providing accurate information for parental counselling and future pregnancy management [22].

An important finding of our study was the identification of several uncommon but highly significant medico-legal circumstances. Maternal physical assault, transplacental amphetamine/methamphetamine exposure, abandoned newborns, unidentified infants, and allegations of medical malpractice were all represented within this relatively small case series [23]. These findings illustrate that forensic investigation of perinatal deaths extends far beyond pathological diagnosis and frequently contributes to judicial decision-making by establishing or excluding causal relationships between maternal events and fetal or neonatal death [24].

### 4.1. Determination of Live Birth in Forensic Investigations

Determining whether an infant was born alive remains one of the primary objectives of forensic investigation because it directly influences criminal responsibility and judicial interpretation. Although the hydrostatic lung flotation test has historically been regarded as an important adjunct in this assessment, contemporary forensic practice recognizes that no single examination is sufficient. Artificial ventilation, decomposition, pulmonary hypoplasia, severe prematurity, or intrauterine gas formation may all produce misleading results. In the present study, live birth determination was based on the integration of hydrostatic testing with gross autopsy findings, histopathological examination, obstetric records, and judicial investigation. This multidisciplinary approach provided greater diagnostic reliability than reliance on any individual method and reflects current international forensic recommendations [25].

### 4.2. Assessment of Fetal Viability

Assessment of fetal viability has important medico-legal implications because viability influences legal interpretation in many jurisdictions. However, viability should not be determined solely according to gestational age. In forensic practice, fetal maturity, birth weight, developmental findings, congenital abnormalities, and maternal clinical history should all be considered together. Several cases in our series required forensic evaluation of viability despite the absence of an identifiable cause of death, demonstrating that forensic opinion frequently extends beyond pathological diagnosis. Standardized assessment of viability therefore represents an essential component of perinatal forensic investigations [26].

### 4.3. Maternal Violence and Causality Assessment

Maternal physical assault during pregnancy presents a particularly challenging forensic scenario because the presence of trauma alone does not establish legal causation. In our series, two cases involved allegations of maternal assault. In one case, placental abruption secondary to blunt trauma was determined to be the direct cause of fetal death, whereas no causal relationship could be established in the second case. These findings illustrate the importance of correlating maternal injuries, placental pathology, fetal autopsy findings, and clinical records before attributing fetal death to traumatic events. Careful causality assessment is essential for both criminal investigations and judicial proceedings [27].

### 4.4. Forensic Implications of Transplacental Drug Exposure

Transplacental transfer of illicit drugs represents an increasingly recognized cause of fetal and neonatal morbidity and mortality. In the present study, one infant died as a consequence of transplacental amphetamine/methamphetamine intoxication, while another demonstrated drug positivity but could not be fully evaluated because of advanced decomposition. These findings highlight the value of routine forensic toxicological analysis whenever maternal substance use is suspected. Identification of fetal drug exposure has important implications not only for determination of the cause of death but also for criminal investigations, child protection, and public health interventions [28].

### 4.5. Investigation of Suspected Medical Negligence

Perinatal deaths occurring in healthcare facilities frequently result in allegations of medical malpractice. In our study, two hospital stillbirths were referred for forensic evaluation following complaints by the families. In such cases, forensic investigation extends beyond autopsy and requires detailed review of obstetric management, fetal monitoring records, timing of clinical interventions, and pathological findings. Independent forensic assessment provides objective scientific evidence for judicial authorities while simultaneously contributing to quality improvement in obstetric care [29].

### 4.6. Identification and Medico-Legal Challenges of Abandoned Newborns

Abandoned or unidentified newborns represent one of the most complex categories of forensic perinatal investigation. Establishing identity, determining whether the infant was born alive, estimating the postmortem interval, and reconstructing the circumstances surrounding death require close cooperation among forensic pathologists, law enforcement agencies, and forensic genetic laboratories. In the present series, abandoned and unidentified infants demonstrated the necessity of integrating autopsy findings with DNA analysis, scene investigation, and police inquiries. These cases emphasize that forensic examination serves not only to establish the cause of death but also to support victim identification and criminal investigation [30].

Placental and umbilical cord examinations also played an important role in selected cases. Placental abruption secondary to maternal trauma and umbilical cord entanglement were identified as direct causes of fetal death. Numerous studies have demonstrated that placental pathology substantially increases the diagnostic yield of perinatal autopsy and frequently provides information that cannot be obtained from fetal examination alone [31]. For this reason, placental examination should be considered an integral component of every forensic investigation of perinatal death whenever available [32].

Despite advances in postmortem investigation, determination of the exact cause of death was not possible in all cases. Advanced decomposition prevented reliable assessment of both live birth status and the mechanism of death in two infants. This finding highlights one of the major limitations of forensic pathology and emphasizes the importance of timely recovery, appropriate preservation of the body, and comprehensive scene investigation to maximize diagnostic accuracy [33]. Six cases remained unexplained despite complete autopsy and routine histopathological examination. Several factors may have contributed to the absence of a definitive diagnosis, including limited placental availability, absence of genomic investigations, lack of specialized neuropathological assessment of autonomic brainstem nuclei, and intrinsic limitations of current forensic diagnostic methods.

Although routine histopathological examination included evaluation of the central nervous system, no structural abnormalities were identified in the examined brain tissue that could explain death in these unexplained intrauterine cases. Nevertheless, detailed neuropathological assessment of the autonomic brainstem nuclei according to current SIUD/SIDS protocols was not routinely performed. Therefore, these unexplained fetal deaths may represent cases that would currently be classified within the spectrum of Sudden Intrauterine Unexplained Death (SIUD) [34]. Future studies incorporating standardized neuropathological protocols together with molecular and genetic investigations may further improve the diagnostic evaluation of unexplained perinatal deaths.

Recent advances in genomic and molecular autopsy have substantially improved the diagnostic yield of unexplained perinatal deaths. Whole-exome sequencing, whole-genome sequencing, and molecular investigations are increasingly recommended as complementary tools when conventional autopsy fails to identify a definitive cause of death [35]. Integration of these techniques into forensic perinatal investigations may allow reclassification of a proportion of currently unexplained cases and improve both diagnostic accuracy and genetic counselling for affected families [36].

**Figure 1:**
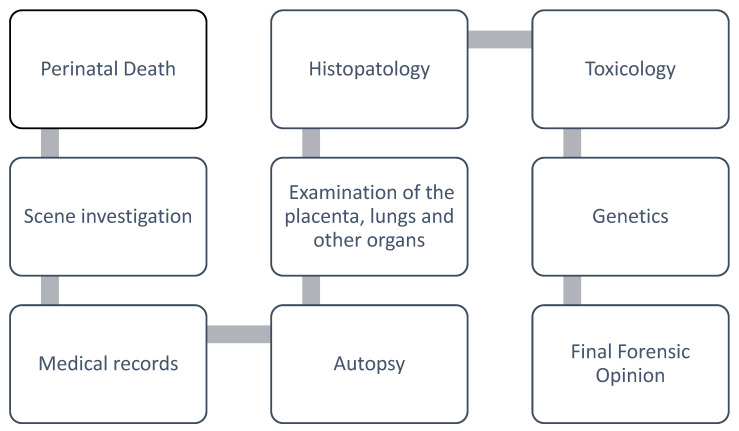
Forensic Investigation Algorithm for Perinatal Deaths.

Our study also highlights the importance of multidisciplinary collaboration. Accurate evaluation of perinatal deaths requires close cooperation among forensic pathologists, obstetricians, neonatologists, pediatric pathologists, radiologists, toxicologists, geneticists, and law enforcement authorities [2]. Such collaboration not only improves diagnostic accuracy but also contributes to the prevention of future perinatal deaths through identification of preventable maternal, fetal, and healthcare-related factors [37]. The algorithm for forensic investigation of perinatal deaths is shown in Figure 1.

## 5. Strengths and Limitations

The principal strength of this study is that it presents a comprehensive medico-legal evaluation of forensic perinatal deaths by integrating autopsy findings with histopathological, toxicological, obstetric, and judicial information. Furthermore, relatively uncommon forensic scenarios—including maternal trauma, transplacental drug exposure, abandoned infants, and suspected medical malpractice—are discussed within the same case series. The algorithm for forensic investigation of perinatal deaths is shown in Figure 1The study has several limitations. First, it is a retrospective single-center study with a relatively limited number of cases. Second, complete placental examination was not available in every case. Third, genetic investigations were unavailable in several fetuses with suspected congenital anomalies. These limitations may have reduced the diagnostic yield in selected cases.

Nevertheless, the findings demonstrate the indispensable role of forensic autopsy in clarifying the cause and manner of perinatal death and underline the importance of a standardized multidisciplinary investigation protocol.

The relatively small number of cases reflects the rarity of perinatal deaths requiring formal medico-legal investigation rather than selective case inclusion. Consequently, our findings should be interpreted as descriptive observations from a forensic case series and should not be generalized to the overall population of perinatal deaths. Nevertheless, the diversity of medico-legal circumstances encountered provides valuable insight into forensic decision-making in this specific setting.

## 6. Conclusions

Perinatal death investigation is one of the most complex areas of forensic medicine, requiring the integration of autopsy findings, obstetric history, maternal medical conditions, histopathological and toxicological examinations, and judicial investigation. The present study demonstrates that forensic evaluation extends beyond determining the immediate cause of death and plays a crucial role in establishing fetal viability, distinguishing live birth from stillbirth, identifying traumatic and non-natural deaths, and evaluating potential medico-legal responsibility.

Our findings highlight the broad spectrum of forensic scenarios encountered in perinatal deaths, including congenital anomalies, prematurity, maternal disease, placental pathology, maternal trauma, transplacental drug exposure, abandoned newborns, and allegations of medical malpractice. These diverse circumstances emphasize that no single diagnostic method is sufficient for establishing the cause and manner of death. Instead, accurate diagnosis requires a multidisciplinary approach combining comprehensive autopsy, histopathological and toxicological investigations, placental and umbilical cord examination, and careful correlation with clinical and investigative findings.

The study also underlines the importance of standardized forensic protocols in improving diagnostic accuracy and ensuring consistency in medico-legal practice. Systematic examination of the fetus or newborn together with the placenta, umbilical cord, and relevant maternal information not only facilitates accurate determination of the cause of death but also provides reliable scientific evidence for judicial authorities and contributes to quality improvement in obstetric and neonatal care.

Although this study is limited by its retrospective design and relatively small number of cases, it demonstrates the value of comprehensive forensic investigation in clarifying perinatal deaths with diverse medico-legal backgrounds. Future multicenter studies with larger case series and routine incorporation of genetic, molecular, and advanced placental examinations may further improve the understanding of perinatal mortality and strengthen forensic diagnostic practice.

In conclusion, forensic autopsy remains the cornerstone of perinatal death investigation. When performed within a multidisciplinary framework and supported by appropriate ancillary investigations, it provides essential information for determining the cause and manner of death, guiding judicial decision-making, and contributing to the prevention of avoidable perinatal deaths.

## Figures and Tables

**Table 1:** Demographic, Clinical Characteristics and Birth Status of the Perinatal Cases.

Case	Sex	Length (cm)	Weight (g)	Gestational Age	Birth Status
1	Male	43	1923	34–35 weeks	Live birth
2	Male	42	2028	33–34 weeks	Live birth
3	Female	53	3230	42 weeks	Live birth
4	Male	41	1343	32–33 weeks	Stillbirth
5	Male	26.5	350	21–22 weeks	Stillbirth
6	Male	46	2260	30–35 weeks	Stillbirth
7	Male	35	1300	27–28 weeks	Live birth
8	Undetermined	51	1966	39–40 weeks	Undetermined
9	Female	45	1877	36–37 weeks	Undetermined
10	Male	42	2370	28–30 weeks	Stillbirth
11	Male	40	2580	36–37 weeks	Stillbirth
12	Male	48	3275	38 weeks	Stillbirth
13	Female	32	508	25–26 weeks	Stillbirth
14	Male	30	1650	23–25 weeks	Stillbirth
15	Male	32	850	24–32 weeks	Stillbirth
16	Male	49	2360	39–40 weeks	Live birth
17	Female	45	2032	32 weeks	Live birth

**Table 2:** Descriptive Statistics of the Perinatal Cases.

Variable	Mean ± SD	Median (IQR)	Minimum	Maximum
Gestational age (weeks)	32.6 ± 6.0	32.5 (28.0–36.5)	21.5	42.0
Birth weight (g)	1876.6 ± 825.0	1966 (1343–2360)	350	3275
Body length (cm)	41.2 ± 7.7	42.0 (35.0–46.0)	26.5	53.0

**Table 3:** Autopsy procedures and ancillary investigations.

Procedure	*n/N* (%)
Full autopsy performed	14/17 (82.4)
External examination only	3/17 (17.6)
Standard anthropometric measurements	17/17 (100)
Toxicological analysis	14/14 (100)
Histopathological examination	14/14 (100)
Placenta available for histopathology	5/14 (35.7)
Umbilical cord examined histologically	13/14 (92.9)
Hydrostatic lung flotation test performed	14/14 (100)
Positive flotation test	4/14 (28.6)
Negative flotation test	8/14 (57.1)
Amphetamine/methamphetamine detected	2/14 (14.3)

**Table 4:** Relationship between live birth status and pulmonary findings in autopsied cases.

Birth Status	Positive Hydrostatic Test/Aerated Lungs	Negative Hydrostatic Test/Unventilated Lungs	No Lung Evaluation
Live birth	4	0	2
Stillbirth	0	8	1
Undetermined	0	0	2

**Table 5:** Final forensic conclusions and causes of death.

Final Conclusion	*n*
Blunt head trauma with skull fracture and intracranial hemorrhage	1
Respiratory failure due to intrauterine pulmonary infection	1
Transplacental amphetamine/methamphetamine intoxication	1
Placental abruption following maternal trauma	1
Respiratory failure due to pulmonary immaturity	1
Polyhydramnios with congenital anomaly	1
Intrauterine asphyxia due to umbilical cord entanglement	1
Intrauterine fetal death following maternal death	1
Neural tube defect	1
Natural death related to underlying disease	1
Intrauterine developmental anomaly	1
Cause of death undetermined	6

## Data Availability

The original contributions presented in this study are included in the article. Further inquiries can be directed to the corresponding author.
